# The Association Between Metabolic Disturbance and Cognitive Impairments in Early-Stage Schizophrenia

**DOI:** 10.3389/fnhum.2020.599720

**Published:** 2021-02-22

**Authors:** Xing-Jie Peng, Gang-Rui Hei, Ran-Ran Li, Ye Yang, Chen-Chen Liu, Jing-Mei Xiao, Yu-Jun Long, Ping Shao, Jing Huang, Jing-Ping Zhao, Ren-Rong Wu

**Affiliations:** ^1^Mental Health Institute of the Second Xiangya Hospital, China National Clinical Research Center on Mental Disorders, China National Technology Institute on Mental Disorders, Hunan Key Laboratory of Psychiatry and Mental Health, Central South University, Changsha, China; ^2^Brain Hospital of Hunan Province, Changsha, China; ^3^Shanghai Institutes for Biological Sciences, Chinese Academy of Sciences, Shanghai, China

**Keywords:** metabolic disturbance, MCCB, early-stage schizophrenia, cognitive impairment, correlation–regression analysis

## Abstract

**Background**: Cognitive impairment is one of the core symptoms of schizophrenia, which is considered to be significantly correlated to prognosis. In recent years, many studies have suggested that metabolic disorders could be related to a higher risk of cognitive defects in a general setting. However, there has been limited evidence on the association between metabolism and cognitive function in patients with early-stage schizophrenia.

**Methods**: In this study, we recruited 172 patients with early-stage schizophrenia. Relevant metabolic parameters were examined and cognitive function was evaluated by using the MATRICS Consensus Cognitive Battery (MCCB) to investigate the relationship between metabolic disorder and cognitive impairment.

**Results**: Generally, the prevalence of cognitive impairment among patients in our study was 84.7% (144/170), which was much higher than that in the general population. Compared with the general Chinese setting, the study population presented a higher proportion of metabolic disturbance. Patients who had metabolic disturbance showed no significant differences on cognitive function compared with the other patients. Correlation analysis showed that metabolic status was significantly correlated with cognitive function as assessed by the cognitive domain scores (*p* < 0.05), while such association was not found in further multiple regression analysis.

**Conclusions**: Therefore, there may be no association between metabolic disorder and cognitive impairment in patients with early-stage schizophrenia.

**Trial Registration**: Clinicaltrials.gov, NCT02880462. Registered August 26, 2016.

## Background

Cognitive impairment is one of the core symptoms of schizophrenia, which refers to dysfunction of attention, working memory, executive function, verbal memory, creativity, and other advanced cognitive functions (Green et al., 2000; Lesh et al., 2011; Guo et al., 2019). These defects persist throughout the course of schizophrenia and are closely related to social dysfunction (Silverstein et al., 1998). So far, antipsychotics, which are recommended as the main treatment for schizophrenia, have demonstrated to have a satisfactory effect on positive symptoms. However, previous studies has shown that antipsychotics may fail to improve cognitive function or have minimal effects on cognitive impairment (Harvey et al., 2001; Mueser and McGurk, 2004). For example, the results from the Clinical Antipsychotic Trials of Intervention Effectiveness (CATIE) indicate that the effects of antipsychotics on cognitive dysfunction are small (Heinrichs, 2007; Keefe et al., 2007). Therefore, more and more psychiatrists are concerned about how to improve the cognitive impairment of patients with schizophrenia (Kahn and Keefe, 2013). However, there is no effective treatment yet since the pathological mechanisms of cognitive impairment are still unclear.

Numerous studies in nonpsychiatric individuals have suggested that metabolic disturbance, including impaired fasting glucose, dyslipidemia, hypertension, and abdominal obesity, could be related to cognitive impairments, which may even induce cognitive dysfunction (Manschot et al., 2007; Assuncao et al., 2018). For instance, type 2 diabetes is recognized as one of the risk factors for cognitive dysfunction (Palta et al., 2014; Liu et al., 2020). Moreover, patients with abdominal obesity are more likely to develop cognitive impairment (Chan et al., 2013; Hou et al., 2019). The same results were found in chronic schizophrenia patients, showing that patients with metabolic disorder have more severe cognitive dysfunction than those without metabolic problems (Boyer et al., 2013; Depp et al., 2014; Li et al., 2014; de Nijs et al., 2016). For example, a study in youth found that patients with metabolic disorder have poorer school performance than those without (de Nijs et al., 2016). Another large sample study also found that attention, immediate memory, and delayed memory in the metabolic syndrome are significantly worse than those in the nonmetabolic syndrome patients (Li et al., 2014). These studies indicate that metabolic disturbance may play an important role in cognitive impairment.

Meanwhile, current research shows that patients with schizophrenia also have a higher risk of metabolic disorders. More than 40% of the schizophrenia patients are diagnosed with metabolic syndrome. About 3–26% of the patients with drug-naive first-episode schizophrenia developed metabolic disorders (McEvoy et al., 2005; Chadda et al., 2013), and it may be associated with a possible common genetic basis for metabolic disorders and schizophrenia (Huang et al., 2010; Liu et al., 2013). Moreover, the risk of metabolic disorders could be further increased after antipsychotic treatment. This study aimed to investigate the association between metabolic status as indicated by relevant parameters and cognitive dysfunction in patients with early-stage schizophrenia.

## Materials and Methods

### Patients and Materials

A total of 172 patients with early-stage schizophrenia admitted to four hospitals in different Chinese provinces from December 2016 to May 2019 were included in the study. Fifty-eight patients were recruited from the Second Xiangya Hospital of Central South University, 40 patients were from the First Affiliated Hospital of Zhengzhou University, 44 patients were from the Second Affiliated Hospital of Xinxiang Medical College, and 30 patients were from the Affiliated Brain Hospital of Guangzhou Medical University. The participants must meet the following criteria: (1) patients were diagnosed based on the Diagnostic and Statistical Manual of Mental Disorders Fourth Edition (DSM-IV); (2) patients were either in their first episodes and had not received any antipsychotic treatment or were acutely ill with an illness duration of less than 36 months. The latter group had received antipsychotics but had not been on these medications for a minimum of 3 months prior to induction; and (3) patients were in the age range between 18 and 50 years. A comprehensive assessment was performed during the screening stage, including physical examination, laboratory tests, personal and family history investigation, and cognitive function assessment. Patients who met any of the following criteria were excluded from our study: (1) substance abuse/dependance; (2) with organic brain disorder; (3) at higher risk of suicide; (4) under treatment with antidepressants, mood stabilizers, and modified electroconvulsive therapy (MECT); (5) comorbidities; (6) pregnant or lactating women; and (7) patients who had administered medication that may impact metabolism, e.g., statins, metformin, and so on.

This study was approved by the Ethics Committee of the Second Xiangya Hospitals, and informed consent was obtained from all the participants prior to study inclusion.

### Clinical Measures

Psychiatric symptoms and the cognitive function of patients were evaluated by several independent experienced psychiatrists by using the Positive and Negative Syndrome Scale (PANSS) and MATRICS Consensus Cognitive Battery (MCCB), respectively. The MCCB consists of nine standardized cognitive tests which reflect seven domains of cognitive function, namely the speed of processing [Trail Making Test: part A (TMT), Brief Assessment of Cognition in Schizophrenia: symbol coding (BACS SC), and category fluency: animal (Animal fluency)], attention/vigilance (Continuous Performance Test, CPT), working memory (digital sequence and Wechsler Memory Scale Spatial Span, WMS III), verbal learning and memory [Hopkins Verbal Learning Test-Revised (HVLT-R)], visual learning and memory (Brief Visuospatial Memory Test-Revised, BVMT-R), reasoning and problem solving (Neuropsychological Assessment Battery, NAB), and social cognition (Mayer–Salovey–Caruso Emotional Intelligence Test, MSCEIT). In the last few years, the MCCB has been translated into Chinese and was widely used in both healthy individuals and schizophrenia patients (Shi et al., 2015, 2019). All cognitive assessments were performed in strict accordance with the instruction manual of the tools by trained personnel. All test scores were adjusted according to Chinese standards for age, gender, and education level and converted into *T*-scores.

When patients were enrolled to the study, their corresponding waist circumference (WC), body mass index (BMI), systolic pressure (SP), and diastolic pressure (DP) were also collected.

### Laboratory Tests

Four blood samples were collected between 8 and 10 a.m. from all patients after fasting overnight on enrolment. Two blood samples were used to measure fasting blood glucose (FBG), triglyceride (TG), low-density lipoprotein cholesterol (LDL-C), high-density lipoprotein cholesterol (HDL-C), and cholesterol (CHO), while the other two samples were centrifugated at 3,000 rpm for 10 min to obtain separate plasma, which were then stored at −80°C for further use. The plasma insulin levels were measured by using enzyme-linked immunosorbent assay (ELISA) with a commercially available kit (Abcam 100578, quantitative). All samples were processed by professional technicians according to the standard instructions provided by the manufacturer.

### Diagnostic Criteria of Metabolic Disturbance

Metabolic disturbance was defined based on the diagnostic criteria which was widely used in China. Patients who met one or more of the following items are considered to have metabolic disturbance: (1) BMI ≥28 kg/m^2^ (Zhou, 2002); (2) WC ≥90/80 cm in males/females (Zhou, 2002); (3) SP ≥140 mmHg and/or DP ≥90 mmHg (World Health Organization, 1999); (4) FBG ≥5.6 mmol/l (Fox et al., 2015); and (5) dyslipidemia: TG ≥1.7 mmol/l, total CHO ≥5.18 mmol/l, HDL-C < 1.04 mmol/l, or LDL-C ≥3.37 mmol/l. The Homeostasis Model Assessment-Insulin Resistance Index (HOMA-IR) was calculated by using the following formula [plasma glucose (mmol/l) * plasma insulin (mIU/l)]/22.5.

### The Severity of Cognitive Dysfunction Based on the Global Deficit Score

The Global Deficit Score (GDS) is used to identify the severity degree of cognitive dysfunction of patients with schizophrenia, which is a sensitive method for the classification of all impairment status (Carey et al., 2004). In our study, all the MCCB test scores were translated into *T*-scores and converted to deficit scores (DS) according to the following criteria: DS = 0 when *T*-score >39 (normal cognitive function), DS = 1 as 35 ≤ *T*-score ≤39 (mild impairment), DS = 2 as 30 ≤ *T*-score ≤34 (mild to moderate impairment), DS = 3 as 25 ≤ *T*-score ≤29 (moderate impairment), DS = 4 as 20 ≤ *T*-score ≤24 (moderate to severe impairment), and DS = 5 as *T*-score < 20 (severe impairment). The final GDS was defined as the average of the DS obtained from each MCCB test. Individuals with a final GDS of ≥0.5 were classified as having cognitive impairment (Taylor and Heaton, 2001; Carey et al., 2004).

### Statistical Analysis

Before analysis, data were tested for distribution normality by using the Shapiro–Wilk test. Differences in the general clinical characteristics, metabolic parameters, and cognitive scores between different groups were compared by using *t* test and analysis of variance (ANOVA) for continuous variables and chi-square test for categorical variables. For the MCCB comparisons between patients with and without abdominal obesity, we included age, gender, duration of illness, and education years as covariates in the multivariate analyses of covariance (MANCOVA). Pearson’s correlation analysis and Spearman correlation analysis were used to investigate the association between the metabolic status indicated by metabolic parameters and cognitive function as assessed by using the MCCB domain scores. Except for TG, FBG, plasma insulin concentration, and HOMA-IR, Pearson correlation analysis was used for the rest of the parameters that satisfy the normal distribution. Metabolic parameters and cognitive scores which displayed significant correlations were selected to be further examined by multiple regression analysis. A multiple linear regression study was required to meet the following assumptions: (1) observations were independent of each other; (2) residual error variance was homogeneous; (3) no multicollinearity; (4) no outliers; and (5) residuals approximated a normal distribution. Gender, age, education years, subcenter, and the duration of illness were included as potential confounders in the regression analysis. Considering that two cohorts of patients were included in our study, indicators that differ between groups would be adjusted in the regression analysis. All statistical analyses were performed with SPSS version 26.0. *p* < 0.05 was considered statistically significant.

## Results

### Demographic and Clinical Characteristics

The general demographic and clinical data of the total number of patients (*n* = 172) are listed in Table 1. A total of 90 drug-naive first-episode schizophrenia patients and 82 patients whose duration of illness was less than 36 months but had used antipsychotics before were included in our study. Comparisons between two groups [drug-naive first-episode schizophrenia patients (FES) and 82 patients whose duration of illness was less than 36 months but had used antipsychotics before] are listed in Supplementary Table S1. The results show that compared with patients who had received antipsychotics, drug-naive first-episode schizophrenia patients have less illness duration (*p* < 0.001), lower BMI (*p* = 0.034), lower LDL-C levels (*p* = 0.007), lower TG levels (*p* = 0.037), and lower CHO levels (*p* = 0.021). However, both SP and DP in drug-naive FES patients were higher than those in other patients (both *p* < 0.05). There is no significant difference between the cognitive test scores and other demographic and clinical characteristics of different groups of patients. For the antipsychotic treatment before enrolment, 39 patients received risperidone, 18 patients used olanzapine, nine patients were on aripiprazole, and 2, 3, and 1 patient, respectively, received sulpiride, quetiapine, and amisulpride. A total of 90 patients never used antipsychotics before, and the data of 10 patients were not clear. All patients had discontinued the above antipsychotic treatment for at least 3 months before enrolment.

**Table 1:** Demographic and clinical characteristics and metabolic parameters of the total number of patients (*n* = 172).

Variables	
Gender (male/female)	76/96
Age^a^ (years)	24.32,6.69
Smoking (have/not)	22/150
Education^a^ (years)	10.95,2.61
Illness duration^a^ (months)	15.03,12.68
Total PANSS^a^	97.01,15.68
PANSS P^a^	22.81,6.47
PANSS N^a^	25.16,6.84
PANSS G^a^	49.04,8.58
BMI^a^ (kg/m^2^)	21.47,3.79
HDL-C levels^a^ (mmol/l)	1.36,0.37
LDL-C levels^a^ (mmol/l)	2.42,0.77
Triglyceride levels^a^ (mmol/l)	1.11,0.73
Cholesterol levels^a^ (mmol/l)	4.08,0.93
Systolic pressure^a^ (mmHg)	113.09,12.00
Diastolic pressure^a^ (mmHg)	72.29,9.87
Waist circumference^a^ (cm)	80.61,11.20
Fasting blood glucose^a^ (mmol/l)	4.76,0.73
Plasma insulin^a^ (μIU/ml)	6.11,3.08
HOMA-IR^a^	1.32,0.90

^a^Data presented as mean ± SD. Note: BMI, body mass index; HDL-C, high-density lipoprotein cholesterol; LDL-C, low-density lipoprotein cholesterol; HOMA-IR, homeostasis model assessment-insulin resistance index calculated as [plasma glucose (mmol/l) * plasma insulin (mIU/L)]/22.5; PANSS P, Positive and Negative Symptom Scale positive score; PANSS N, Positive and Negative Symptom Scale negative score; PANSS G, Positive and Negative Symptom Scale general pathology score; Total PANSS, Positive and Negative Symptom Scale total score.

### Comparisons Between Cognitive Function Across Early-Stage Schizophrenia and Healthy Controls

The domain scores of the MCCB test are shown in Table 2. Compared with the *T*-scores of the standard cognitive performance of 656 healthy adults in China (Shi et al., 2015, 2019), patients with early-stage schizophrenia had significantly lower scores in seven domains, which indicated cognitive dysfunction (all *p* < 0.001). According to the GDS, 84.7% (144/170) of the patients were defined as having cognitive impairment, and the proportion was higher than that in the general population (16.5%).

**Table 2:** Comparing the cognitive scores from the MATRICS Consensus Cognitive Battery (MCCB) in early-stage schizophrenia and healthy adults.

	Patients (*n* = 170)	Controls^a^ (*n* = 656)	*p*-value
TMT	36.80 ± 12.06	50.00 ± 10.00	<0.001***
BACS SC	29.74 ± 11.61	50.00 ± 10.00	<0.001***
HVLT-R	33.89 ± 11.43	50.00 ± 10.00	<0.001***
WMS III	37.34 ± 12.83	50.00 ± 10.00	<0.001***
NAB	38.97 ± 12.33	50.00 ± 10.00	<0.001***
BVMT-R	39.53 ± 13.32	50.00 ± 10.00	<0.001***
CPT	37.98 ± 12.42	50.00 ± 10.00	<0.001***
Animal fluency	37.71 ± 11.85	50.00 ± 10.00	<0.001***
MSCEIT	37.47 ± 11.74	50.00 ± 10.00	<0.001***
Speed of processing	34.77 ± 9.12	50.00 ± 10.00	<0.001***
Attention vigilance	37.98 ± 12.42	50.00 ± 10.00	<0.001***
Working and memory	37.34 ± 12.83	50.00 ± 10.00	<0.001***
Verbal learning and memory	33.89 ± 11.43	50.00 ± 10.00	<0.001***
Visual learning and memory	39.53 ± 13.32	50.00 ± 10.00	<0.001***
Reasoning and problem solving	38.97 ± 12.33	50.00 ± 10.00	<0.001***
Social cognition	37.47 ± 11.74	50.00 ± 10.00	<0.001***
GDS			
GDS < 0.50	15.3%	83.5%	
0.5 < GDS ≤ 1	16.5%	13.6%	
1 < GDS ≤ 2	34.7%	2.6%	
2 < GDS ≤ 3	20.0%	0.3%	
3 < GDS ≤ 4	11.2%	0%	
4 < GDS ≤ 5	2.3%	0%	
GDS ≥ 5	0%	0%	

Note: two patients enrolled in our study lack MCCB performance score. TMT, Trail Making Test: part A; BASC SC, Brief Assessment of Cognition in Schizophrenia: symbol coding; HVLT-R, Hopkins Verbal Learning Test-Revised; WMS II, Digital Sequence and Wechsler Memory Scale spatial span; NAB, Neuropsychological Assessment Battery; BVMT-R, Brief Visuospatial Memory Test-Revised; CPT, Continuous Performance Test; Animal fluency, category fluency: animal; MSCEIT, Mayer–Salovey–Caruso Emotional Intelligence Test; GDS, Global Deficit Score. ^a^Normative standards of China were based on Shi et al. (2015) that was published before which included 656 healthy controls. ***p < 0.001.

### Metabolic Status in Early-Stage Schizophrenia

The metabolic parameters are shown in Table 1. A total of 109 (63.4%) patients are defined as having metabolic disturbance based on the criteria. Furthermore, 10 (5.8%) patients had obesity (BMI ≥ 28 kg/m^2^), 21 (12.4%) subjects had hypertension (SP ≥ 140 mmHg and/or DP ≥ 90 mmHg), 59 (34.3%) patients had abdominal obesity (WC ≥ 90/80 cm in male/female), 14 (8.4%) had impaired fasting glucose (FBG ≥ 5.6 mmol/l), 2 (1.2%) subjects had diabetes (FBG ≥ 7.0 mmol/l), and 5 (3.1%) patients had insulin resistance (HOMA-IR ≥ 2.69). There were 62 (37.8%) patients having at least one abnormality in the levels of LDL-C, HDL-C, CHO, and triglycerides, which was considered as dyslipidemia. In summary, a total of 13 (8.3%) patients were determined to have metabolic syndrome according to the diagnostic criteria of the International Diabetes Federation (IDF).

### Comparisons of Cognitive Function Across Early-Stage Schizophrenia With and Without Metabolic Disturbance

Patients in this study were divided into two groups depending on whether or not they met the criteria of metabolic disturbance. Compared with patients without metabolic disturbance, patients with metabolic disturbance showed a significant higher performance score on the TMT (*p* = 0.03). There are no significant differences in other MCCB test scores or domain scores between the two groups (all *p* > 0.05).

### Correlation Analysis Between Metabolic Parameters and Cognitive Scores in Early-Stage Schizophrenia

**Figure 1:**
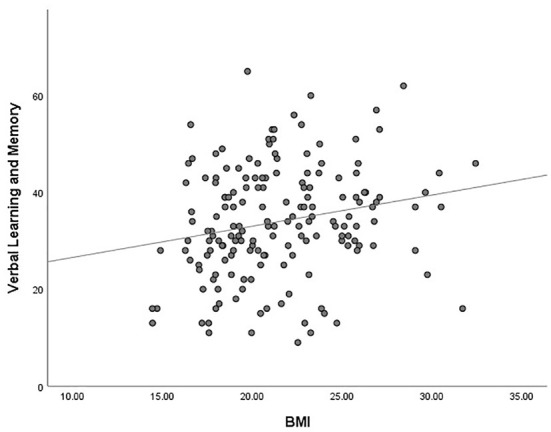
MATRICS Consensus Cognitive Battery (MCCB) verbal learning and memory score was positively associated with body mass index (BMI) of the total number of study patients (*p* = 0.007).

**Figure 2:**
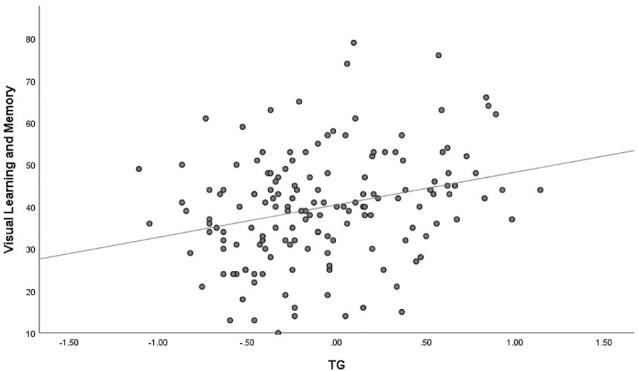
MCCB visual learning and memory score was positively associated with triglyceride (TG) of the total number of study patients (*p* < 0.001).

The outlier which was three times higher or lower than the standard error was excluded from the analysis, in order to reflect the true correlation between metabolic parameters and cognitive domain scores. The results of correlation analysis between metabolic parameters and cognitive scores are presented in Table 3. Verbal learning and memory capability were found to be significantly positively correlated to BMI (*r* = 0.21, *p* = 0.007, Figure 1). A positive correlation was also found between TG levels and capability of visual learning and memory (*r* = 0.284, *p* < 0.001, Figure 2). Other metabolic parameters including BMI, CHO levels, and SP were also, respectively, associated with working memory, speed of processing and reasoning, and problem solving (all *p* < 0.05). In addition, WC was associated with multiple domains of cognitive function including attention/vigilance, working memory, and verbal learning and memory as well as visual learning and memory (all *p* < 0.05). However, no association was found between cognitive function and SP or FBG (all *p* > 0.05).

**Table 3:** Correlation analysis on metabolic parameters and cognitive scores in early-stage schizophrenia patients (part of all).

	Speed of processing		Attention vigilance		Working memory		Verbal learning and memory		Visual learning and memory		Reasoning and problem solving		Social cognition	
	*r*	*p*	*r*	*p*	*r*	*p*	*r*	*p*		*p*	*r*	*p*	*r*	*p*
BMI^a^	0.085	0.277	–0.070	0.378	0.182	0.018^*	0.207	0.007**	0.137	0.080	0.116	0.134	–0.046	0.567
TG^b^	0.156	0.052	0.075	0.359	0.132	0.099	0.188	0.018^*	0.284	<0.001***	0.128	0.112	0.095	0.248
CHO^a^	0.177	0.025^*	–0.069	0.397	0.111	0.163	0.054	0.494	0.025	0.753	0.004	0.960	0.061	0.454
SP^a^	0.017	0.824	0.151	0.057	0.035	0.652	–0.044	0.569	0.091	0.248	0.173	0.025^*	0.056	0.488
WC^a^	0.028	0.720	–0.163	0.040^*	0.195	0.012^*	0.164	0.034^*	0.173	0.028^*	0.089	0.255	–0.059	0.460
Insulin^a^	–0.046	0.565	0.145	0.076	0.006	0.936	–0.034	0.669	0.012	0.879	–0.140	0.078	0.199	0.015^*
HOMA-IR^b^	0.009	0.908	0.059	0.480	0.068	0.403	0.010	0.906	0.067	0.413	–0.104	0.201	0.195	0.019^*

^a^Used Pearson correlation analysis. ^b^Used Spearman correlation analysis. *p < 0.05; **p < 0.01; ***p < 0.001.

### Multiple Regression on Metabolic Parameters and Cognitive Test Scores in Early-Stage Schizophrenia

Indicators showing significant association in the above correlation analysis were studied for further multiple regression analysis. Assumptions for multiple linear regressions were met. The results of multiple regression are presented in Table 4. The results of the regression analysis show that HDL-C levels (*β* = −0.239, *p* = 0.004, 95% CI, −13.196 to −2.504) are independent negative predictors for TMT after adjusted for gender, age, education years, subcenter and duration of illness. Interestingly, we also found that age (*β* = 0.184, *p* = 0.020, 95% CI, 0.052–0.610) is a positive predictor of TMT. Moreover, TG levels (*β* = 0.220, *p* = 0.011, 95% CI, 1.424–10.870) are positive independent predictors for BVMT-R after adjusted for gender, age, education years, subcenter, duration of illness, and groups. However, no significant indicator was found for cognitive domain scores from the MCCB in multiple regression analysis.

**Table 4:** Multiple regression on metabolic parameters and cognitive test scores in early-stage schizophrenia patients.

	Regression age			HDL-C levels			TG levels		
	*β*	*p*	95% CI	*β*	*p*	95% CI	*β*	*p*	95% CI
TMT	0.184	0.020*	0.052, 0.610	−0.239	0.004**	−13.196, −2.504	–	–	–
BVMT-R	0.145	0.095	−0.049, 0.611	–	–	–	0.220	0.011*	1.424, 10.870

Note: *p < 0.05; **p < 0.01.

## Discussion

This study tried to explore the association between metabolic disturbance and cognitive dysfunction in patients with early-stage schizophrenia.

In this study, the main finding was that we did not find any association between metabolic disturbance and cognitive dysfunction in patients with early-stage schizophrenia. Contrary to a previous hypothesis, the correlation analysis showed that the disturbance of metabolic parameters including increased BMI and WC, dyslipidemia, and elevated SP may have a positive impact on cognitive function. After adjusting for age, sex, duration of illness, education years, groups, and subcenter, further multiple regression analysis showed that HDL-C levels were independent predictors for TMT, and TG levels were independent predictors for animal fluency. However, the results of the regression analysis found that although some metabolic parameters were independently predictive of some cognitive test scores, the metabolic parameters were not predictive for any cognitive domains in early-stage schizophrenia. Moreover, this was the main finding which was contrary to previous studies. In a previous study, Friedman et al. (2010) found that hypertension exerted a significant negative effect on immediate delayed and recognition memory in patients with schizophrenia. Recently, a small-scale study also reported that two domains of the MCCB were associated with glucose levels in patients with early-stage schizophrenia (Zhang et al., 2019). There are several possible explanations for the opposite results. First, the severity of metabolic disorders may have different influences on cognitive function. Therefore, mild metabolic disorders in our study may have no significant effect on cognitive function in patients with early-stage schizophrenia. Second, the publication bias may also highlight positive conclusions. Third, adequate nutrition is the necessary basis for neurodevelopment and cognitive function (Klimová and Vališ, 2018). In the treatment of acute stage, patients may consume more nutrients after antipsychotic treatment, thereby promoting the improvement of cognitive function. Notably, numerous studies in chronic schizophrenia have reported a significant negative correlation between metabolic disorders and cognitive impairment, especially in patients with severe metabolic syndrome (Li et al., 2014; Bora et al., 2018). Moreover, several studies have been done to investigate the potential underlying mechanisms of metabolic syndrome and cognitive impairment, and it was found that insulin resistance could be a key factor of the association between metabolic disorders and cognitive dysfunction (Kim and Feldman, 2015; Zilliox et al., 2016; Kothari et al., 2017). For example, decreased expression of insulin receptors and reduced insulin signaling can be observed in patients with insulin resistance, which can lead to decreased levels of GluN2B and GluA1 phosphorylation at synapses, ultimately resulting in impaired synaptic plasticity and cognitive function (Zilliox et al., 2016). This finding suggests that cognitive function may not be associated with metabolism when patients do not show severe metabolic disorder, and the association between metabolic disorder and cognitive impairment may only occur in comorbidity with severe glucose disturbance.

Several other findings emerged from this study. A trend of dyslipidemia in patients who have taken antipsychotics before has been found as compared with drug-naive FES, including LDL-C levels, TG levels, and CHO levels. This result may be explained by the antipsychotic treatment, especially second-generation antipsychotics which have an influence on the lipid levels (Lindenmayer et al., 2003; Tschoner et al., 2009).

We also found that 84.7% of the patients with early-stage schizophrenia had cognitive impairment as indicated by the cognitive performance score covering seven different domains of MCCB, which is consistent with previous studies (Bozikas et al., 2006; Zanelli et al., 2019; Zhang et al., 2019). These results show that cognitive impairment may occur very early in the course of schizophrenia. Moreover, we found no differences between drug-naive FES and patients who received antipsychotics before in terms of cognitive function. However, for the lipid levels, an obvious distinction between these two groups was found. This conclusion coincides with the results from our regression analysis. Moreover, in our study, female patients with schizophrenia have worse cognition dysfunction than males with respect to working memory, reasoning, and problem solving. This result is consistent with the finding of Pérez-Garza et al. (2016), but it is in contrary to the conclusions of Zhang et al. (2017). Therefore, sex differences in the cognitive impairment of patients with schizophrenia remain unclear.

In addition, our study showed that 63.7% of the patients had metabolic disturbance, while 34.7% of the patients had abdominal obesity, 37.8% had dyslipidemia, and 12.4% had hypertension. The prevalence rates of certain metabolic disturbance were a little bit higher than those in the general population. There have been several evidences that suggested that patients with schizophrenia were susceptible to metabolic disorder, although the mechanism was complicated and unclear. It has been suspected that both schizophrenia itself and antipsychotic medications could contribute to the development of metabolic disorder. Thus, regular monitoring is needed for patients who were diagnosed with metabolic disorders to prevent worsening of metabolic issues.

There are some limitations to be noted in our study. First, we did not collect healthy controls for the comparison of the cognitive function and metabolism of patients with early-stage schizophrenia. Second, some patients in our study were treated with different antipsychotics. Though, we have excluded a patient who had antipsychotics in 3 months before enrolment and we adjusted antipsychotic treatment in our regression analysis, however, different antipsychotic treatment may also have different effects on cognitive function and metabolism. Third, except for WC, we did not have adequate sample size to explore the relationship between abnormal metabolic parameters and cognitive function in patients with metabolic disorders. Therefore, our conclusions on the association between metabolic disorders and cognitive function in patients with early-stage schizophrenia should be interpreted with caution, especially for patients with severe metabolic syndrome. Fourth, the cross-sectional design of this study failed to examine the temporal relationship between cognitive function and metabolic disorders. Fifth, some data were missing due to few patients not completing the test which may affect the results. Sixth, the concept of “early-stage schizophrenia” is different from other studies, though we had defined it very clearly in the “Materials and Methods” section. Finally, we did not adjust for study site basis, which may also impose influences on cognitive test scores.

## Conclusion

In summary, this report confirmed that, in China, the prevalence rates of cognitive impairment and metabolic disturbance in patients with early-stage schizophrenia were higher than those of the general population. Patients receiving antipsychotic treatment have a significant higher impairment of lipid levels compared with drug-naive FES patients. Moreover, there is no difference in the cognitive function between patients with and without metabolic disturbance. In addition, correlation analysis and further multiple regression analysis showed that certain metabolic parameters may be positively correlated to cognitive function, although no metabolic parameter was found as the independent predictor for cognitive domain in our study. Considering the limitations of our study, well-designed longitudinal studies are needed in the future to verify our conclusions.

## Data Availability Statement

The raw data supporting the conclusions of this article will be made available by the authors, without undue reservation.

## Ethics Statement

The studies involving human participants were reviewed and approved by the National Health and Medical Research Council of the Second Xiangya Hospital Ethics Committee. The patients/participants provided their written informed consent to participate in this study. Written informed consent was obtained from the individual(s) for the publication of any potentially identifiable images or data included in this article.

## Author Contributions

X-JP analyzed and interpreted the patient data and was a major contributor in writing the manuscript. G-RH mainly designed and performed the study. YY, C-CL, J-MX, Y-JL, PS, JH, and R-RL helped in patient recruitment, monitoring of data quality, and document treatment emergent adverse events. J-PZ guided the study design. R-RW was responsible for the overall content. All authors contributed to the article and approved the submitted version.

## Conflict of Interest

The authors declare that the research was conducted in the absence of any commercial or financial relationships that could be construed as a potential conflict of interest.

## Abbreviations

MCCB: MATRICS Consensus Cognitive Battery
CATIE: Clinical Antipsychotic Trials of Intervention Effectiveness
DSM-IV: Diagnostic and Statistical Manual of Mental Disorders fourth edition
MECT: modified electroconvulsive therapy
PANSS: Positive and Negative Syndrome Scale
TMT: Trail Making Test: part A
BACS SC: Brief Assessment of Cognition in Schizophrenia: symbol coding
CPT: Continuous Performance Test
WMS III: digital sequence and Wechsler Memory Scale spatial span
BVMT-R: Brief Visuospatial Memory Test-Revised
NAB: Neuropsychological Assessment Battery
MSECIT: Mayer–Salovey–Caruso Emotional Intelligence Test
WC: waist circumference
BMI: body mass index
SP: systolic pressure
DP: diastolic pressure
FBG: fasting blood glucose
TG: triglyceride
LDL-C: low-density lipoprotein cholesterol
HDL-C: high-density lipoprotein cholesterol
CHO: cholesterol
ELISA: enzyme-linked immunosorbent assays
HOMA-IR: Homeostasis Model Assessment-Insulin Resistance Index
GDS: Global Deficit Score
ANOVA: analysis of variance
MANCOVA: multivariate analysis of covariance
FES: first-episode schizophrenia.
