# Corrigendum: The association between metabolic disturbance and cognitive impairments in early-stage schizophrenia

**DOI:** 10.3389/fnhum.2022.1094810

**Published:** 2022-11-21

**Authors:** Xing-Jie Peng, Gang-Rui Hei, Ran-Ran Li, Ye Yang, Chen-Chen Liu, Jing-Mei Xiao, Yu-Jun Long, Ping Shao, Jing Huang, Jing-Ping Zhao, Ren-Rong Wu

**Affiliations:** ^1^Mental Health Institute of the Second Xiangya Hospital, China National Clinical Research Center on Mental Disorders, China National Technology Institute on Mental Disorders, Hunan Key Laboratory of Psychiatry and Mental Health, Central South University, Changsha, China; ^2^Brain Hospital of Hunan, Changsha, China; ^3^Shanghai Institutes for Biological Sciences, Chinese Academy of Sciences, Shanghai, China

**Keywords:** metabolic disturbance, MCCB, early-stage schizophrenia, cognitive impairment, correlation–regression analysis

In the published article, there was an error in the **Trial Registration**. Instead of “NCT03451734. Registered March 2, 2018 (retrospectively registered)” it should be “NCT02880462. Registered August 26, 2016.”

The authors apologize for this error and state that this does not change the scientific conclusions of the article in any way. The original article has been updated.

## Publisher's note

All claims expressed in this article are solely those of the authors and do not necessarily represent those of their affiliated organizations, or those of the publisher, the editors and the reviewers. Any product that may be evaluated in this article, or claim that may be made by its manufacturer, is not guaranteed or endorsed by the publisher.

