# Supplementary material for: The Association Between Metabolic Disturbance and Cognitive Impairments in Early-Stage Schizophrenia
Source: Front Hum Neurosci. 2021 Feb 22;14:599720. doi: 10.3389/fnhum.2020.599720 (PMC7937877; doi:10.3389/fnhum.2020.599720)
Supplement: Supplementary file 1 [file Data_Sheet_1.docx]

**Table S1.** **Comparing the general clinical characteristics, metabolic parameters and cognitive scores from two subjects’ groups (90** **drug naïve patients and 82 patients with history of acute illness farther than 3 months ago)**

**Table S2.** **Comparing the cognitive scores from the MATRICS Consensus Cognitive Battery in early-stage schizophrenia with and without metabolic disturbance**

**Table S3.** **Correlation analysis on metabolic parameters and cognitive scores in early-stage schizophrenia (All).**

**Table S1. Comparing the general clinical characteristics, metabolisms and cognitive scores in drug naïve patients and patients with history of acute illness farther than 3 months ago.**

|  | Group 1  *N* = 90 | | | Group 2  *N* = 82 | | | | t/ X^2^ | P |
| --- | --- | --- | --- | --- | --- | --- | --- | --- | --- |
|  | *N*  % | | | N % | | | |  | |
| Male | 35 | | 38.9 | 29 | | | 35.4 | 0.032 | 0.857 |
|  | Mean | | SD | Mean | | | SD |  | |
| Age | 23.79 | | 6.61 | 24.90 | | | 6.77 | -1.091 | 0.277 |
| BMI | 20.89 | | 3.56 | 22.11 | | | 3.94 | -2.136 | 0.034* |
| Education (years) | 11.04 | | 2.54 | 10.85 | | | 2.69 | 0.478 | 0.633 |
| Illness duration (months) | 10.57 | | 10.99 | 19.92 | | | 12.68 | -5.142 | <0.001*** |
| Total PANSS | 98.61 | | 13.81 | 95.26 | | | 17.43 | 1.406 | 0.162 |
| PANSS P | 23.60 | | 6.09 | 21.94 | | | 6.80 | 1.691 | 0.093 |
| PANSS N | 25.19 | | 7.16 | 25.13 | | | 6.51 | 0.052 | 0.958 |
| PANSS G | 49.82 | | 7.50 | 48.18 | | | 9.59 | 1.254 | 0.211 |
| HDL levels (mmol/L) | 1.39 | | 0.38 | 1.32 | | | 0.35 | 1.266 | 0.207 |
| LDL levels (mmol/L) | 2.27 | | 0.67 | 2.59 | | | 0.83 | -2.742 | 0.007** |
| TG levels (mmol/L) | 1.04 | | 0.75 | 1.20 | | | 0.73 | -2.100 | 0.037* |
| CHO levels (mmol/L) | 3.91 | | 0.86 | 4.25 | | | 0.98 | -2.335 | 0.021* |
| SP (mmHg) | 118.64 | | 13.51 | 114.06 | | | 11.00 | 2.410 | 0.017* |
| DP (mmHg) | 76.03 | | 9.85 | 72.13 | | | 8.39 | 2.765 | 0.006** |
| WC (cm) | 80.08 | | 10.84 | 81.19 | | | 11.61 | -0.64 | 0.523 |
| Plasma insulin (μIU/ml) | 5.66 | | 1.34 | 6.57 | | | 4.13 | -0.443 | 0.658 |
| HOMA-IR | 1.17 | | 0.32 | 1.48 | | | 1.23 | -1.400 | 0.163 |
| TMT | 36.06 | | 12.11 | 37.62 | | | 12.03 | -0.842 | 0.401 |
| BACS SC | 30.64 | | 11.14 | 28.75 | | | 12.09 | 1.059 | 0.291 |
| HVLT-R | 33.03 | | 11.60 | 34.84 | | | 11.24 | -1.029 | 0.305 |
| WMS III | 36.38 | | 12.44 | 38.41 | | | 13.24 | -1.028 | 0.306 |
| NAB | 38.53 | | 11.85 | 39.46 | | | 12.90 | -0.491 | 0.624 |
| BVMT-R | 38.91 | | 13.83 | 40.25 | | | 12.76 | -0.644 | 0.521 |
| CPT | 38.03 | | 12.04 | 37.91 | | | 12.93 | 0.065 | 0.948 |
| Animal fluency | 37.24 | | 12.44 | 38.25 | | | 11.22 | -0.554 | 0.580 |
| MSCEIT | 38.10 | | 12.05 | 36.76 | | | 11.42 | 0.715 | 0.476 |
| Speed of processing | 34.64 | | 9.15 | 34.91 | | | 9.14 | -0.189 | 0.850 |
| Attention/Vigilance | 38.03 | | 12.04 | 37.91 | | | 12.93 | 0.065 | 0.948 |
| Working and memory | 36.38 | | 12.44 | 38.41 | | | 13.24 | -1.028 | 0.306 |
| Verbal learning/Memory | 33.03 | | 11.60 | 34.84 | | | 11.24 | -1.029 | 0.305 |
| Visual learning/Memory | 38.91 | | 13.83 | 40.25 | | | 12.76 | -0.644 | 0.521 |
| Reasoning/Problem solving | 38.53 | | 11.85 | 39.46 | | | 12.90 | -0.491 | 0.624 |
| Social cognition | 38.10 | | 12.05 | 36.76 | | | 11.42 | 0.715 | 0.476 |
| GDS | *N* | % | | *N* | % | | |  | |
| GDS < 0.50 | 10 | 11.2 | | 16 | | 19.8 | | 2.375 | 0.123 |
| 0.5 < GDS ≤ 1 | 20 | 22.5 | | 14 | | 17.3 | |  |  |
| 1 < GDS ≤ 2 | 37 | 41.6 | | 23 | | 28.4 | |  |  |
| 2 < GDS ≤ 3 | 11 | 12.4 | | 21 | | 25.9 | |  |  |
| 3 < GDS ≤ 4 | 10 | 11.2 | | 6 | | 7.4 | |  |  |
| 4 < GDS ≤ 5 | 1 | 1.1 | | 1 | | 1.2 | |  |  |
| GDS ≥ 5 | 0 | | | 0 | | | |  |  |
| *Note:* Group 1= 90 drug naïve patients, Group 2 = 82 patients with history of acute illness farther than 3 months ago. Both two groups have one patient missing cognitive scores data.  The Plasma insulin, TG levels and HOMA-IR are not normally distributed and were log-transformed to approximate a normal distribution when analysis.  PANSS P: Positive and negative symptom scale positive score;  PANSS N: Positive and negative symptom scale negative score;  PANSS G: Positive and negative symptom scale General Pathology score;  Total PANSS: Positive and negative symptom scale total score.  *p < 0.05 **p < 0.01 ***p < 0.001 | | | | | | | | | |

**Table S2. Comparing the cognitive scores from the MATRICS Consensus Cognitive Battery in early-stage schizophrenia with and without metabolic disturbance**

|  | Metabolic disturbance (n=108) | Non-metabolic disturbance (n=62) | P value |
| --- | --- | --- | --- |
| TMT | 38.32±12.38 | 34.15±11.07 | 0.03* |
| BACS SC | 30.63±11.39 | 28.19±11.91 | 0.19 |
| HVLT-R | 34.58±10.80 | 32.69±12.47 | 0.30 |
| WMS III | 37.87±13.57 | 36.44±11.48 | 0.49 |
| NAB | 38.67±13.07 | 39.48±11.01 | 0.68 |
| BVMT-R | 39.65±12.76 | 39.33±14.34 | 0.88 |
| CPT | 38.32±12.30 | 37.36±12.72 | 0.64 |
| Animal fluency | 38.08±11.86 | 37.08±11.92 | 0.60 |
| MSCEIT | 37.78±12.22 | 36.89±10.89 | 0.65 |
| Speed of processing | 35.72±8.97 | 33.14±9.21 | 0.08 |
| Attention Vigilance | 38.32±12.30 | 37.36±12.72 | 0.64 |
| Working and memory | 37.87±13.57 | 36.44±11.48 | 0.49 |
| Verbal learning and memory | 34.58±10.80 | 32.69±12.47 | 0.30 |
| Visual learning and memory | 39.65±12.76 | 39.33±14.34 | 0.88 |
| Reasoning and problem solving | 38.67±13.07 | 39.48±11.01 | 0.68 |
| Social cognition | 37.78±12.22 | 36.89±10.89 | 0.65 |
| Data presented as mean±SD  *p < 0.05 **p < 0.01 ***p < 0.001 | | | |

| **Table S3.** **Correlation analysis on metabolic parameters and cognitive scores in early-stage schizophrenia (All)..** | | | | | | | | | | | | | | | | | |
| --- | --- | --- | --- | --- | --- | --- | --- | --- | --- | --- | --- | --- | --- | --- | --- | --- | --- |
|  |  | TMT | BACS SC | HVLT-R | WMS III | NAB | BVMT-R | Animal Fluency | MSCEIT | CPT IP | Processing speed | Attention/Vigilance | Working memory | Verbal Learning | Visual Learning | Reasoning/  Problem solving | Social Cognition |
| BMI | Pearson | 0.142 | 0.002 | .207^**^ | .182^*^ | 0.116 | 0.137 | 0.047 | -0.046 | -0.070 | 0.085 | -0.070 | .182^*^ | .207^**^ | 0.137 | 0.116 | -0.046 |
|  | Sig | 0.066 | 0.983 | 0.007 | 0.018 | 0.134 | 0.080 | 0.546 | 0.567 | 0.378 | 0.277 | 0.378 | 0.018 | 0.007 | 0.080 | 0.134 | 0.567 |
| FBG | Spearman | -0.032 | 0.027 | 0.005 | 0.098 | -0.022 | 0.028 | 0.122 | 0.075 | 0.009 | 0.037 | 0.009 | 0.098 | 0.005 | 0.028 | -0.022 | 0.075 |
|  | Sig | 0.686 | 0.733 | 0.951 | 0.216 | 0.777 | 0.723 | 0.123 | 0.356 | 0.914 | 0.641 | 0.914 | 0.216 | 0.951 | 0.723 | 0.777 | 0.356 |
| HDL-C | Pearson | -.201^*^ | 0.090 | -0.031 | -0.085 | -0.134 | -0.122 | 0.145 | -0.028 | -0.059 | 0.010 | -0.059 | -0.085 | -0.031 | -0.122 | -0.134 | -0.028 |
|  | Sig | 0.010 | 0.254 | 0.691 | 0.283 | 0.089 | 0.126 | 0.068 | 0.730 | 0.470 | 0.897 | 0.470 | 0.283 | 0.691 | 0.126 | 0.089 | 0.730 |
| LDL-C | Pearson | 0.008 | 0.098 | 0.026 | 0.120 | 0.021 | 0.010 | .239^**^ | 0.131 | -0.035 | 0.152 | -0.035 | 0.120 | 0.026 | 0.010 | 0.021 | 0.131 |
|  | Sig | 0.923 | 0.218 | 0.744 | 0.132 | 0.793 | 0.903 | 0.003 | 0.109 | 0.672 | 0.056 | 0.672 | 0.132 | 0.744 | 0.903 | 0.793 | 0.109 |
| TG | spearman | 0.131 | .163^*^ | .188^*^ | 0.132 | 0.128 | .284^**^ | 0.098 | 0.095 | 0.075 | 0.156 | 0.075 | 0.132 | .188^*^ | .284^**^ | 0.128 | 0.095 |
|  | Sig | 0.101 | 0.040 | 0.018 | 0.099 | 0.112 | 0.000 | 0.225 | 0.248 | 0.359 | 0.052 | 0.359 | 0.099 | 0.018 | 0.000 | 0.112 | 0.248 |
| CHO | Pearson | -0.020 | .180^*^ | 0.054 | 0.111 | 0.004 | 0.025 | .247^**^ | 0.061 | -0.069 | .177^*^ | -0.069 | 0.111 | 0.054 | 0.025 | 0.004 | 0.061 |
|  | Sig | 0.797 | 0.022 | 0.494 | 0.163 | 0.960 | 0.753 | 0.002 | 0.454 | 0.397 | 0.025 | 0.397 | 0.163 | 0.494 | 0.753 | 0.960 | 0.454 |
| SP | Pearson | -0.048 | 0.053 | -0.044 | 0.035 | .173^*^ | 0.091 | 0.038 | 0.056 | 0.151 | 0.017 | 0.151 | 0.035 | -0.044 | 0.091 | .173^*^ | 0.056 |
|  | Sig | 0.538 | 0.496 | 0.569 | 0.652 | 0.025 | 0.248 | 0.631 | 0.488 | 0.057 | 0.824 | 0.057 | 0.652 | 0.569 | 0.248 | 0.025 | 0.488 |
| DP | Pearson | -0.013 | 0.129 | 0.021 | -0.002 | 0.070 | 0.115 | 0.026 | -0.032 | -0.051 | 0.062 | -0.051 | -0.002 | 0.021 | 0.115 | 0.070 | -0.032 |
|  | Sig | 0.869 | 0.096 | 0.787 | 0.983 | 0.371 | 0.143 | 0.743 | 0.689 | 0.524 | 0.428 | 0.524 | 0.983 | 0.787 | 0.143 | 0.371 | 0.689 |
| WC | Pearson | 0.126 | -0.078 | .164^*^ | .195^*^ | 0.089 | .173^*^ | 0.015 | -0.059 | -.163^*^ | 0.028 | -.163^*^ | .195^*^ | .164^*^ | .173^*^ | 0.089 | -0.059 |
|  | Sig | 0.106 | 0.317 | 0.034 | 0.012 | 0.255 | 0.028 | 0.853 | 0.460 | 0.040 | 0.720 | 0.040 | 0.012 | 0.034 | 0.028 | 0.255 | 0.460 |
| Insulin | spearman | -0.078 | -0.048 | -0.034 | 0.006 | -0.140 | 0.012 | 0.011 | .199^*^ | 0.145 | -0.046 | 0.145 | 0.006 | -0.034 | 0.012 | -0.140 | .199^*^ |
|  | Sig | 0.326 | 0.547 | 0.669 | 0.936 | 0.078 | 0.879 | 0.889 | 0.015 | 0.076 | 0.565 | 0.076 | 0.936 | 0.669 | 0.879 | 0.078 | 0.015 |
| HOMA-IR | spearman | -0.043 | 0.019 | 0.010 | 0.068 | -0.104 | 0.067 | 0.072 | .195^*^ | 0.059 | 0.009 | 0.059 | 0.068 | 0.010 | 0.067 | -0.104 | .195^*^ |
|  | Sig | 0.600 | 0.813 | 0.906 | 0.403 | 0.201 | 0.413 | 0.376 | 0.019 | 0.480 | 0.908 | 0.480 | 0.403 | 0.906 | 0.413 | 0.201 | 0.019 |

Note: *p < 0.05 **p < 0.01 ***p < 0.001
